# Prognostic value, immune signature and molecular mechanisms of the SUMO family in pancreatic adenocarcinoma

**DOI:** 10.3389/fmolb.2022.1096679

**Published:** 2022-12-15

**Authors:** Yunjie Duan, Yongxing Du, Yongrun Mu, Zongting Gu, Chengfeng Wang

**Affiliations:** ^1^ State Key Lab of Molecular Oncology and Department of Pancreatic and Gastric Surgery, National Cancer Center/National Clinical Research Center for Cancer/Cancer Hospital, Chinese Academy of Medical Sciences and Peking Union Medical College, Beijing, China; ^2^ Department of Hepatobiliary and Pancreatic Surgery and Minimally Invasive Surgery, Zhejiang Provincial People’s Hospital, Hangzhou Medical College, Hangzhou, Zhejiang, China; ^3^ Shanxi Province Cancer Hospital/ Shanxi Hospital Affiliated to Cancer Hospital, Chinese Academy of Medical Sciences/Cancer Hospital Affiliated to Shanxi Medical University, Taiyuan, Shanxi, China

**Keywords:** pancreatic adenocarcinoma, SUMO family, TP53, M6A, biomarkers, prognosis, immune infiltration

## Abstract

**Background:** Pancreatic adenocarcinoma (PAAD) has a high degree of malignancy and a very poor prognosis, and the 5-year overall survival rate of patients is approximately 7%. To improve the prognosis of patients with PAAD, a more comprehensive and in-depth study of the pathogenesis of PAAD and the identification of new diagnostic markers and treatment targets are urgently needed. Increasing evidence supports that the small ubiquitin-like modifier (SUMO) family is closely related to the occurrence and development of a variety of cancers. However, the function of the SUMO family in PAAD is not clear, and related research is very scarce.

**Methods:** R, Cytoscape, cBioPortal, and other software and online databases were used to comprehensively analyze the expression characteristics, prognostic value, and oncogenic mechanism of the SUMO family in PAAD.

**Results:** SUMO family members are highly expressed in PAAD, and high expression of SUMO family members is significantly associated with poor clinicopathological features and poor prognosis in PAAD patients. In addition, SUMO family members are significantly coexpressed with M6A methylation regulators and various oncogenes and play an activating role in various oncogenic pathways, including EMT. Furthermore, it is worth noting that the close association between SUMO family members and TP53 mutation status and the negative regulatory effect of SUMO1/2 on PAAD immunity may represent the potential mechanism by which SUMO family members promote the development of PAAD. Moreover, the coexpression characteristics of SUMO family members and a variety of cancer-promoting immune checkpoint genes, as well as the positive correlation between SUMO4 expression level and the sensitivity of various targeted or chemotherapeutic drugs, including gemcitabine, paclitaxel, and doxorubicin, suggest future clinical directions of this study.

**Conclusion:** The SUMO family is closely related to the occurrence and development of PAAD and can be used as a new biomarker and therapeutic target for patients with PAAD.

## Introduction

PAAD is prone to distant metastasis, with a high degree of malignancy and poor prognosis, and the 5-year survival rate is only 7.2% (He et al., 2022). Despite great advances in the treatment of PAAD over the past decades, regrettably, the incidence and mortality of this disease have increased worldwide, especially in countries such as the United States, China, and Brazil (Ali et al., 2021; Chaves et al., 2022; Yin et al., 2022). Recent projections suggest that by 2030, PAAD could surpass colorectal cancer as the second leading cause of cancer-related death after lung cancer (Ali et al., 2021). Surgical resection is the only radical treatment available for PAAD. However, due to the aggressive nature of PAAD and the lack of early symptoms, fewer than 20% of these tumors are resectable at the time of diagnosis (Fan et al., 2020). Therefore, diagnosis and treatment in the early stage of disease are critical to improving the prognosis of PAAD patients. Developing these tools requires a more comprehensive and in-depth study of the pathogenesis of PAAD and the identification of new diagnostic markers and therapeutic targets.

Epigenetics is defined as heritable changes in gene expression that are not accompanied by changes in DNA sequence (Omura and Goggins, 2009), mainly manifested as DNA methylation and histone posttranslational modifications, such as acetylation, methylation, phosphorylation, ubiquitination, glycosylation, and other amino acid modifications (Huo et al., 2021). Normal epigenetic modifications can be altered by the oncogene metabolic reprogramming that occurs in the context of cancer cell proliferation, metastasis and heterogeneity, and abnormal regulation of epigenetic modifications in cancer cells typically plays an important role in metabolism and oxidation–reduction as it affects the biological macromolecular synthesis and energy production processes involved in the development of cancer (Huo et al., 2021). SUMOylation, an epigenetic modification process in which the small ubiquitin-like modifier peptide covalently modifies a protein at a lysine residue (Xu et al., 2020), is carried out predominantly by the SUMO family, which consists of four members: SUMO1, SUMO2, SUMO3 and SUMO4 (Lorente et al., 2019). SUMO family members have different functions in different tumor tissues and cell types. For example, selective inhibition of SUMO1 can induce G1 phase arrest, thereby inhibiting the formation and progression of glioblastoma (Bellail et al., 2014), and METTL3 can increase the metastatic potential of liver cancer when modified by SUMO1 (Xu et al., 2020). SUMO2 promotes the proliferation, migration, and invasion of liver cancer cells by promoting the activities of MMP-9 and vascular endothelial growth factor (Chen et al., 2021), and its overexpression can also enhance the proliferation and metastasis of non-small cell lung cancer cells (Li et al., 2021). Upregulation of SUMO3 is closely associated with the occurrence of acute megakaryoblastic leukemia (Haemmerling et al., 2012), and SUMO4 is closely associated with type I and type II diabetes mellitus (Wang and She, 2008; Pu et al., 2012). In conclusion, an increasing number of SUMOylation proteins have been shown to be highly expressed in tumor tissues, and SUMOylation has become an important posttranslational modification that regulates cellular processes and cancer progression (Xu et al., 2020). Unfortunately, although many studies have indicated an oncogenic role for SUMO family members in different tumors, the roles of these proteins in PAAD are still unclear, and relevant studies are very scarce. Therefore, we conducted a bioinformatic analysis of the functions of SUMO family members in PAAD.

In this study, we performed a variety of bioinformatics analyses of publicly available data to comprehensively investigate the possible mechanisms by which SUMO family members might be involved in PAAD occurrence and development. We analyzed the differences in the mRNA and protein expression of SUMO family members between PAAD tissues and adjacent tissues (and normal pancreatic tissues) for the first time and further analyzed the expression characteristics of SUMO family members at the single-cell level in PAAD tissues. In addition, our study revealed the relationships between the expression levels of SUMO family members and clinicopathological features, TP53 mutation status, the promoter methylation levels of SUMO family members, and the survival time of PAAD patients. The possible roles of SUMO family members in the occurrence and development of PAAD and their potential value in the clinical treatment of PAAD were explored for the first time through gene variation, immune infiltration, gene enrichment, protein‒protein interaction (PPI), and drug sensitivity analyses. Finally, we found that SUMO family members can be used as prognostic biomarkers and novel therapeutic targets for PAAD patients.

## Methods and materials

### The inclusion criterion of PAAD patients for the study

To be qualified for the study, the following requirements were needed to be met by the patients: 1) aged between 18 and 80 years, 2) pathologically proven adenocarcinoma histology, and 3) available follow-up clinical data. Finally, a total of 178 patients fulfilled the inclusion criteria and were included in the final analyses.

### Expression analysis

Based on PAAD project data in the TCGA database and pancreas project data in the GTEx database, the expression levels of SUMO family members in cancer tissues, adjacent tissues, and normal pancreatic tissues were analyzed using R (version 3.6.3). The Wilcoxon test was used for statistical analysis, and *p* < 0.05 was considered statistically significant. Then, the immunohistochemical staining characteristics of SUMO family members in PAAD tissues were analyzed in the HPA database (https://www.proteinatlas.org/). According to staining intensity and quantification, the protein expression levels were classified into four categories: not detected, low, medium, and high (Uhlén et al., 2015). In addition, using the published PAAD single-cell dataset (GSM4679532) (Lin et al., 2020), we analyzed the expression characteristics of SUMO family members at the single-cell level using R (version 3.6.3).

### Analysis of clinicopathological features and prognosis

Using the PAAD project data in the TCGA database, the relationships between the expression levels of SUMO family members and the clinicopathological features of PAAD patients were analyzed using R (version 3.6.3), with statistical analysis performed by Welch’s T-test. *p* < 0.05 was considered statistically significant. The Kaplan‒Meier Plotter database (http://www.kmplot.com/) was used to analyze the correlation between the expression levels of SUMO family members and OS and RFS in PAAD patients (Lánczky and Győrffy, 2021). Log-rank *p* values were calculated using the “survival” package in R (version 2.38), and *p* < 0.05 was considered statistically significant. ROC curves were analyzed and plotted using the “pROC” R package (version 1.17.0.1) and the “ggplot2” R package (version 3.3.3). The area under the ROC curve (AUC) values were between 0.5 and 1.

### Analysis of gene variation and oncogenic mechanisms

The cBioPortal database (http://www.cbioportal.org/) was used to analyze the gene variation characteristics of SUMO family members in PAAD and their relationships with selected clinicopathological features. The chi-square test was used for statistical analysis, and *p* < 0.05 was considered statistically significant (Gao et al., 2013). Using on the TIMER database (http://timer.cistrome.org/), the expression correlation between SUMO family members and m6A methylation regulators, as well as among SUMO family members, were analyzed. Statistical analysis was carried out by Spearman’s test, and *p* < 0.05 was considered statistically significant (Li et al., 2017). The UALCAN database (http://ualcan.path.uab.edu) was used to analyze the correlation between SUMO family member expression levels and promoter methylation levels and TP53 mutation status. Statistical analysis was performed by Welch’s T-test, and *p* < 0.05 was considered statistically significant (Chandrashekar et al., 2017). Then, the LinkedOmics database (http://www.linkedomics.org/) was used to screen the 400 genes whose expression profiles were most similar to those of the SUMO family members (Vasaikar et al., 2018). The Metascape database (https://metascape.org) was used to visualize biological process (BP), cellular components (CC), molecular function (MF), and Kyoto Encyclopedia of Genes and Genomes (KEGG) of SUMO family members and their 400 coexpressed genes (Zhou et al., 2019). Then, oncogenic pathway enrichment analysis of SUMO family members was performed using the GSCALite database (http://bioinfo.life.hust.edu.cn/web/GSCALite/), and the STRING database (https://string-db.org/) and Cytoscape (version 3.9.1) were used to construct the functional network of the genes with the strongest PPIs with SUMO family members and score the effect intensities of these genes (Shannon et al., 2003; Von Mering et al., 2003; Liu et al., 2018).

### Immune infiltration and drug sensitivity analysis

The TISIDB database (http://cis.hku.hk/TISIDB/) was used to analyze the correlations between the expression levels of SUMO family members and the levels of tumor-infiltrating immune cells and the expression levels of immune molecules in PAAD. Spearman’s test was used for statistical analysis, and *p* < 0.05 was considered statistically significant (Ru et al., 2019). The TIMER database was used to analyze the correlations between the changes in the copy numbers of SUMO family members and the infiltration levels of 6 types of immune cells in PAAD. Statistical analysis was performed by the Wilcoxon rank-sum test, and *p* < 0.05 was considered statistically significant (Li et al., 2017). In addition, the TISIDB database was also used to analyze the expression levels of SUMO family members in different immune subtypes of PAAD. The Kruskal‒Wallis test was used for statistical analysis, and *p* < 0.05 was considered statistically significant (Ru et al., 2019). Then, R (version 3.6.3) was used to analyze the correlations of expression levels between SUMO family members and multiple immune checkpoint genes. Spearman’s test was used for statistical analysis, and *p* < 0.05 was considered statistically significant. Finally, the relationships between the expression level of SUMO4 and sensitivity to many kinds of chemotherapy or targeted drugs were analyzed with the GSCALite database. Spearman’s test was used for statistical analysis, and *p* < 0.05 was considered statistically significant (Liu et al., 2018).

## Results

### Abnormal expression of SUMO family members in tumors

**FIGURE 1:**
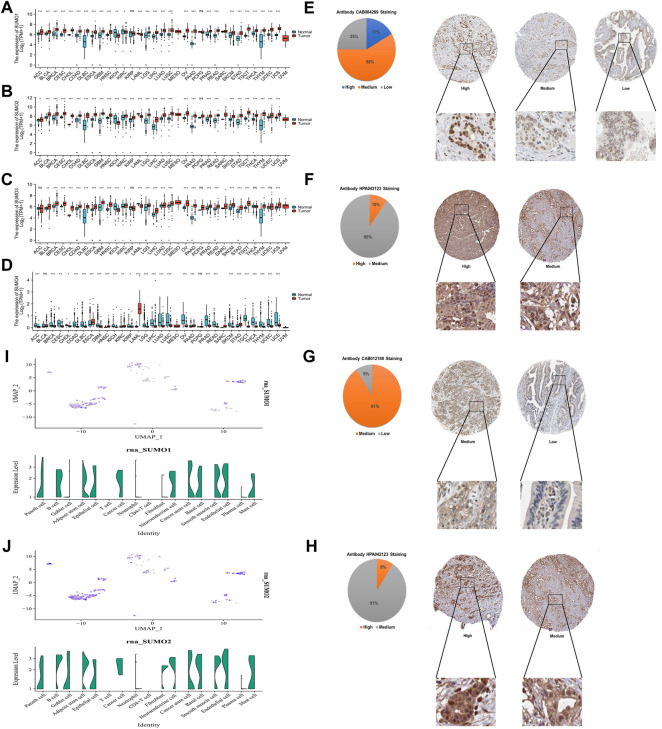
Expression characteristics of SUMO family at the mRNA level in pan-cancer and the protein level in PAAD, and expression characteristics of SUMO1/2 at the single-cell level in PAAD. **(A)** The mRNA level of SUMO1; **(B)** The mRNA level of SUMO2; **(C)** The mRNA level of SUMO3; **(D)** The mRNA level of SUMO4. ns, *p* ≥ 0.05; *, *p* < 0.05; **, *p* < 0.01; ***, *p* < 0.001; **(E)** The protein level of SUMO1; **(F)** The protein level of SUMO2; **(G)** The protein level of SUMO3; **(H)** The protein level of SUMO4; **(I)** The single-cell level of SUMO1; **(J)** The single-cell level of SUMO2.

To explore the expression characteristics of SUMO family members in tumor tissues and corresponding non-tumor tissues at the mRNA level, we used R software (version 3.6.3) to analyze data from The Cancer Genome Atlas (TCGA) database (PAAD project) and Genotype-Tissue Expression (GTEx) database (pancreas project). The results showed that the mRNA expression levels of SUMO1/2/3 in 22 kinds of tumor tissues, including PAAD, stomach adenocarcinoma (STAD), and breast invasive carcinoma (BRCA), were higher than the levels in the corresponding non-tumor tissues, while the mRNA expression levels in kidney chromophobe (KICH) and acute myeloid leukemia (LAML) were lower than those in the corresponding non-tumor tissues (Figures 1A–C). In contrast, the mRNA expression levels of SUMO4 in 22 tumor tissues, including colon adenocarcinoma (COAD), liver hepatocellular carcinoma (LIHC), and ovarian serous cystadenocarcinoma (OV), were lower than those in the corresponding non-tumor tissues; interestingly, however, the SUMO4 mRNA expression level in PAAD was still higher than that in the corresponding non-tumor tissues (Figure 1D). Then, HPA database analysis revealed the protein expression characteristics of SUMO family members in PAAD: the immunohistochemical staining intensity of SUMO family members in PAAD tissues was mostly high or medium (Figures 1E–H). The analysis based on the single-cell PAAD dataset showed that the expression level of SUMO1/2/3 was higher in cancer cells or cancer stem cells and lower in many kinds of tumor-infiltrating immune cells, with the exception of B cells (Figures 1I, J) (Supplementary Figure S1B). In summary, the expression levels of SUMO family members are significantly higher in PAAD than in corresponding non-tumor tissues, suggesting that these factors may play an important role in the occurrence and development of PAAD.

### Relationships between SUMO family member expression levels and clinicopathological features and prognosis in patients with PAAD

**FIGURE 2:**
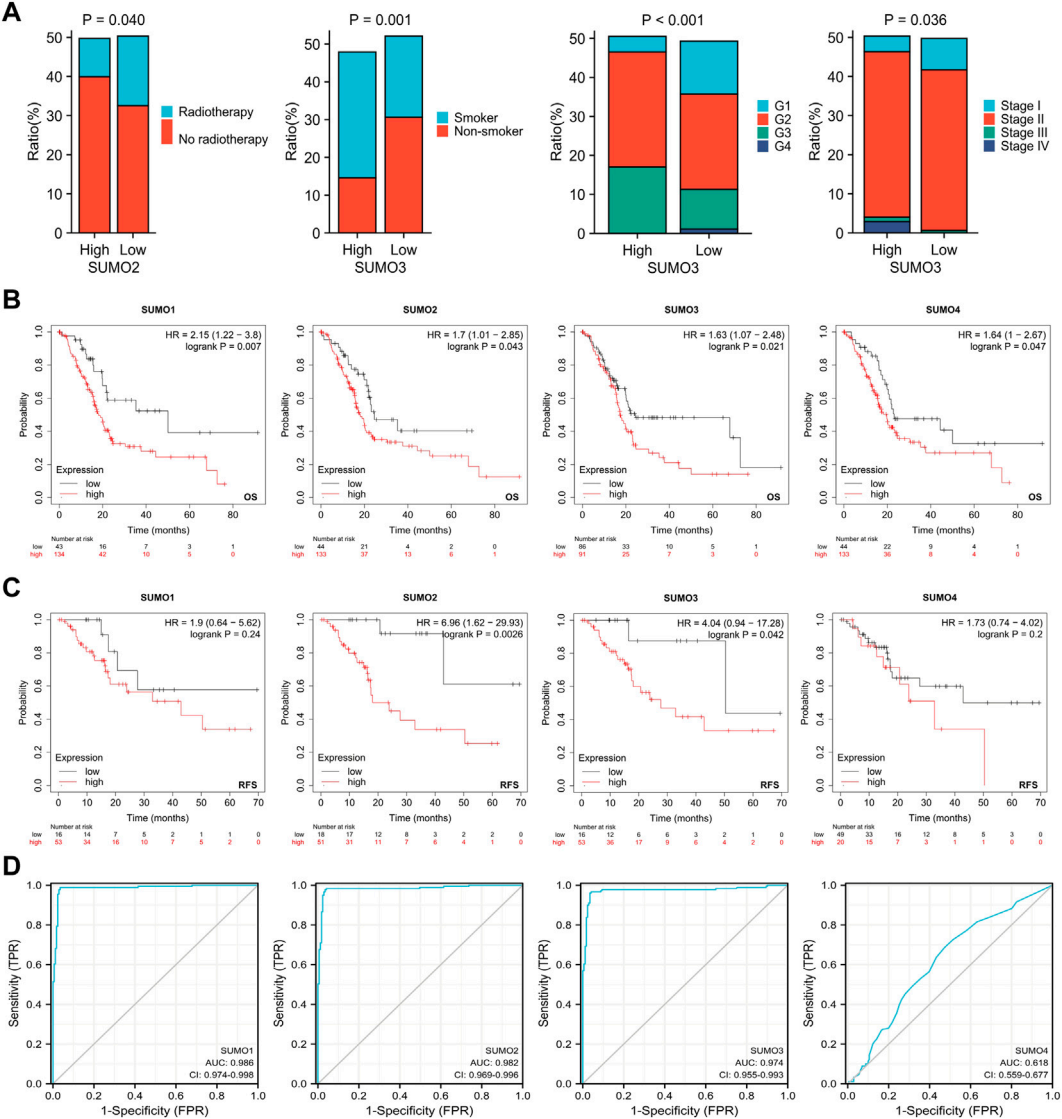
Relationship between expression levels of SUMO family members and clinicopathological features and prognosis of patients with PAAD. **(A)** Higher proportion of PAAD patients with high SUMO2 expression who did not receive radiotherapy; PAAD patients with high SUMO3 expression had a higher proportion of patients with smoking history, higher tumor grade and higher tumor stage; **(B)** The increased expression levels of SUMO family members were significantly correlated with shorter OS; **(C)** The increased expression levels of SUMO 2/3 were significantly correlated with shorter RFS; **(D)** SUMO family members showed high accuracy in predicting normal and neoplastic outcomes.

To explore the relationships between the expression levels of SUMO family members and the clinicopathological characteristics of PAAD patients, we used R software (version 3.6.3) to analyze the relevant data from the TCGA database. The results showed that there was a higher proportion of patients who did not receive radiotherapy among the PAAD patients with high expression of SUMO2 (Figure 2A) (Supplementary Material S1), while there was a higher proportion of patients with a history of smoking, higher tumor grade, and higher tumor stage among the PAAD patients with high expression of SUMO3 (Figure 2A) (Supplementary Material S1). Kaplan‒Meier Plotter database analysis revealed the relationships between the expression levels of SUMO family members and the prognosis of PAAD patients. Higher expression levels of SUMO family members were significantly correlated with shorter overall survival (OS) (Figure 2B), and higher expression levels of SUMO2/3 were also significantly correlated with shorter recurrence-free survival (RFS) (Figure 2C). Finally, we drew ROC curves according to the data from the TCGA and GTEx databases (Figure 2D). The AUC values of SUMO family members were high, indicating that normal pancreatic tissue could be accurately distinguished from PAAD tissue on the basis of SUMO family member expression. In summary, the high expression of SUMO family members in PAAD patients is related to specific clinicopathological features and poor prognosis, suggesting that SUMO family members may promote the occurrence and development of PAAD.

### Genetic variation profiles of SUMO family members in PAAD and their relationship with M6A methylation regulators and TP53 mutation status

**FIGURE 3:**
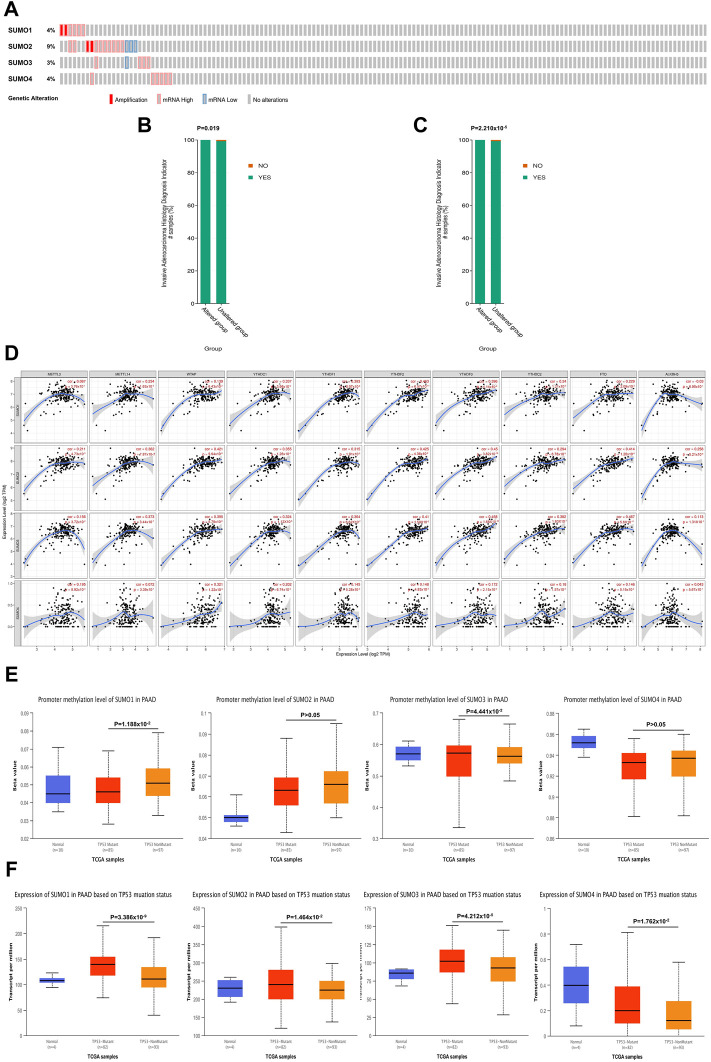
Genetic variation characteristics of SUMO family members in PAAD and relationship of their expression levels with m6A methylation regulators and TP53 mutation status. **(A)** Genetic variation characteristics of the SUMO family members in PAAD; **(B,C)** The amplification and mRNA High mutation of SUMO1/2 was significantly correlated with PAAD invasion of surrounding tissues; **(D)** SUMO family members were positively correlated with the expression levels of most M6A methylation regulators; **(E)** The promoter methylation level of SUMO1 in the TP53 mutant PAAD tissue was significantly lower than that in the TP53 wild-type PAAD tissue, but SUMO3 was the opposite; **(F)** The expression levels of SUMO family members in the TP53 mutant PAAD tissue was significantly higher than that in the TP53 wild-type PAAD tissue.

Using the cBioPortal database, we investigated the genetic variations in SUMO family members in PAAD. SUMO family members had gene mutations in 26 PAAD patient samples (17%). The main mutation type was amplification and mRNA-high, and the gene with the highest mutation frequency was SUMO2 (9%) (Figure 3A). In addition, mutations in SUMO1 (Figure 3B) and SUMO2 (Figure 3C) may be associated with more aggressive PAAD tumors. One particularly important finding was that there were positive correlations between SUMO family members and the expression levels of most M6A methylation regulators (Figure 3D). Another interesting finding was that the expression levels of SUMO family members in TP53 mutant PAAD tissues were higher than those in TP53 wild-type tissues (Figure 3F). In addition, TP53 mutation led to a decrease in the level of SUMO1 promoter methylation and an increase in the level of SUMO3 promoter methylation (Figure 3E). The above findings indicate that the variants of SUMO family members in PAAD are typically amplifications, which may lead to their elevated expression and further lead to a worse prognosis for PAAD patients. However, due to the low proportion of patients with mutations, this finding needs to be further verified in a large sample. In addition, TP53 mutation leads to increased expression of SUMO family members, suggesting that SUMO family members may be downstream targets regulated by TP53, and the overexpression of SUMO family genes may increase the expression levels of M6A methylation regulators with cancer-promoting effects. This may be a potential mechanism by which SUMO family members promote the occurrence and development of PAAD.

### Enrichment analysis of SUMO family members and 400 coexpressed genes

**FIGURE 4:**
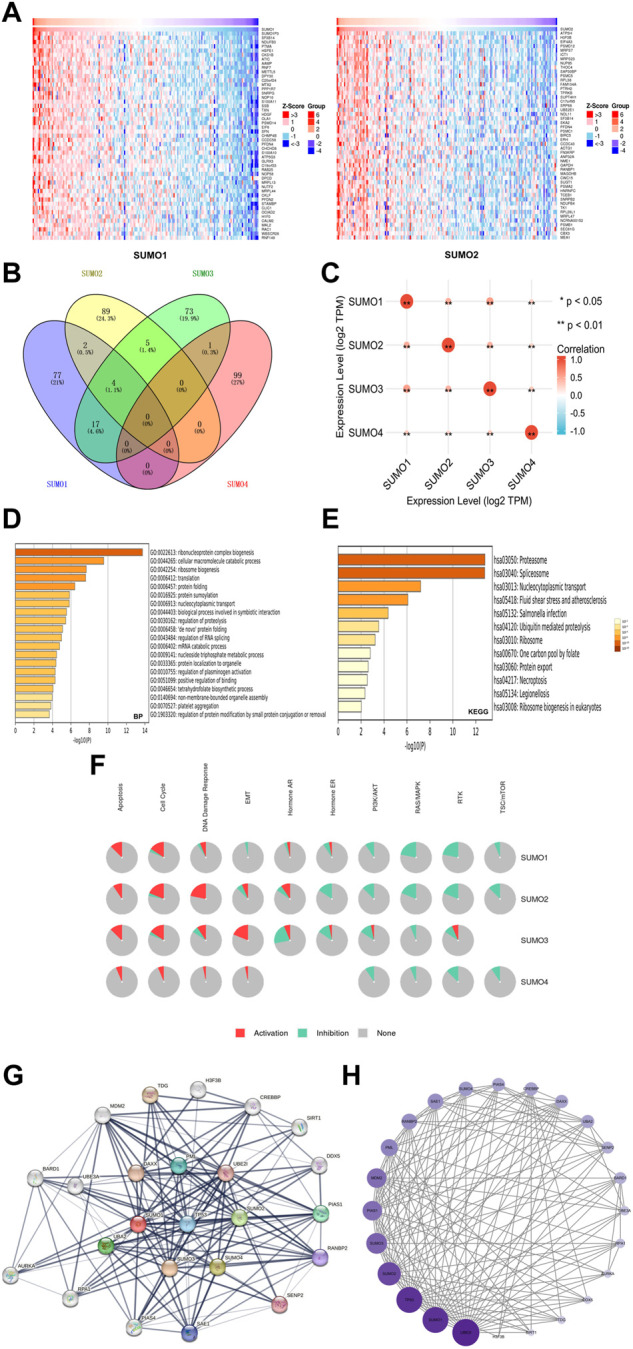
Gene enrichment analysis and PPI network construction of SUMO family in PAAD. **(A)** Top 50 genes co-expressed with SUMO1/2; **(B)** The venn diagram of SUMO family members and their 400 co-expressed genes; **(C)** SUMO family members are also co-expressed among themselves. *, *p* < 0.05; **, *p* < 0.01; **(D)** The GO enrichment of the BP terms of the SUMO family and its 400 co-expressed genes; **(E)** The KEGG enrichment of the SUMO family and its 400 co-expressed genes; **(F)** SUMO family played an activating role in a variety of oncogenic pathways; **(G,H)** TP53 played an important role in PPI network which was closely related to SUMO family.

To further explore the mechanisms by which SUMO family members may impact the occurrence and development of PAAD, we used the LinkedOmics database to identify the 400 genes that were most closely coexpressed with SUMO family members. The top 200 genes are shown in a heatmap (Figure 4A) (Supplementary Figure S2). Four genes, SF3B14, PFDN4, PPIA, and TCEB1, were coexpressed with SUMO1/2/3 (Figure 4B) (Supplementary Material S3). In addition, all SUMO family members were coexpressed with one another (Figure 4C) (Supplementary Material S4). Then, we performed GO and KEGG enrichment analyses of the SUMO family members and their 400 coexpressed genes using the Metascape database (Supplementary Material S5). GO analysis showed that this set of genes was mainly enriched in “ribonucleoprotein complex biogenesis”, “cellular macromolecule catabolic process”, “ribosome biogenesis” and other BP terms (Figure 4D), “catalytic step 2 spliceosome”, “endopeptidase complex”, “ficolin-1-rich granule lumen” and other CC terms (Supplementary Figure S3A) and “unfolded protein binding”, “cadherin binding”, “structural constituent of the ribosome” and other MF terms (Supplementary Figure S3B). KEGG analysis showed that the SUMO family members and their 400 coexpressed genes were mainly enriched in pathways related to “proteasome”, “spliceosome” and “nucleocytoplasmic transport” (Figure 4E). On the basis of these findings, we further analyzed the activities of SUMO family members on various oncogenic pathways using the GSCALite database. The SUMO family members were found to have major activating effects on “Apoptosis”, “Cell Cycle”, and “DNA Damage Response” pathways, as shown in Figure 4F. SUMO3/4 were also found to play an activating role in the epithelial–mesenchymal transition (EMT) pathway. These studies indicate that SUMO family members are closely related to the translation of protein-coding genes and posttranslational modifications of proteins. In addition, the coexpression characteristics of SUMO family members with various oncogenes, including TCEB1, and their activation of various oncogenic pathways may suggest another potential mechanism through which these proteins may promote the occurrence and development of PAAD.

### Construction and analysis of the SUMO family member PPI network

To construct and analyze the PPI network of SUMO family members in PAAD patients, we identified the 24 genes with the strongest PPIs with SUMO family members using the STRING database (Supplementary Material S6). The associated PPI network was then plotted using Cytoscape software (version 3.9.1), with larger circles and darker colors indicating a larger number of PPIs associated with the gene. The results showed that TP53 was located at the core of the SUMO family PPI network (Figure 4G) and had a strong influence on other genes (Figure 4H) (Supplementary Material S7). These results suggest that TP53 plays an important role in the PPI network involving the SUMO family members. The above findings may provide additional evidence that SUMO family members are regulated by TP53.

### Immune landscape of SUMO family members in PAAD

**FIGURE 5:**
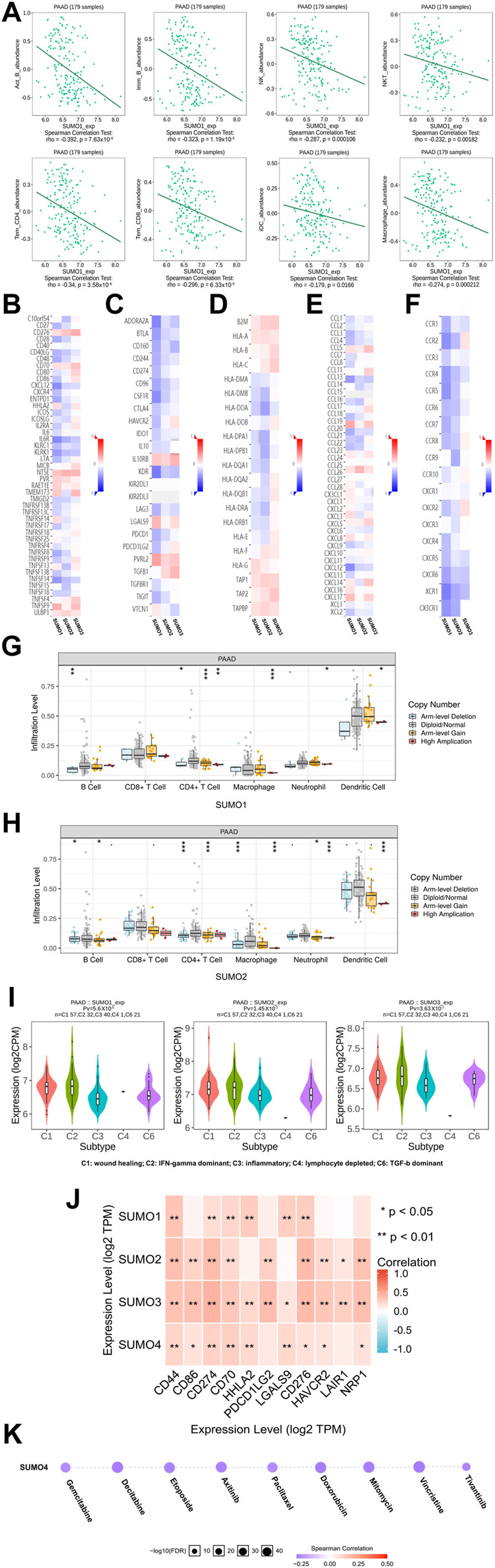
The immune landscape of SUMO family in PAAD. **(A)** The expression level of SUMO1 was negatively correlated with the infiltration level of a variety of tumor infiltrating immune cells, including Act B, Imm B and Tem CD8; **(B–F)** The expression level of SUMO1/2 was negatively correlated with most immunomodulators, but SUMO3 was the opposite; **(G,H)** The copy-number alteration of SUMO1/2 may affect the infiltration levels of six types of tumor infiltrating immune cells. *, *p* < 0.05; **, *p* < 0.01; ***, *p* < 0.001; **(I)** The expression of SUMO 1/2/3 was significantly different among the five immune subtypes; **(J)** There are significant co-expression correlations between SUMO family members and several immune checkpoint genes, including CD44, HHLA2 and HAVCR2. *, *p* < 0.05; **, *p* < 0.01; **(K)** The expression of SUMO4 was positively correlated with the sensitivity of various targeting or chemotherapeutic drugs, including gemcitabine, paclitaxel, and doxorubicin.

To explore the immunological characteristics associated with SUMO family members in PAAD, we analyzed the relationships between the expression of SUMO family members and the presence of tumor-infiltrating immune cells, various immunomodulators, and various immune markers in the PAAD project data in the TISIDB database. Although SUMO4 data are not included in these databases, our findings reveal strikingly different regulatory roles for SUMO1/2/3 in tumor immunity. First, the expression levels of SUMO1/2 were negatively correlated with the infiltration levels of a variety of tumor-infiltrating immune cells, including Act B, Imm B, and Tem CD8 T cells (Figure 5A) (Supplementary Figures S4A, B). In addition, the SUMO1/2 expression level was inversely correlated with that of most immune modulators (immune promoters, MHC molecules, chemokines, and chemokine receptors) (Figures 5B, C–F). Therefore, high expression of SUMO1/2 may inhibit the immune response to PAAD. In contrast, the expression level of SUMO3 was positively correlated with the tumor infiltration levels of various immune cells and with the expression of most immune modulators (Supplementary Figure S4C) (Figures 5B, D–F). This finding indicates that SUMO3 may be an important factor in enhancing the immune response to PAAD.

Then, we explored the relationship between copy number alterations in SUMO family members and the levels of tumor-infiltrating immune cells in data from the PAAD project in the TIMER database. The results showed that changes in the copy number of SUMO1/2 may affect the infiltration levels of 6 tumor-infiltrating immune cells, especially Arm-level Gain and High Application (Figures 5G,H) (Supplementary Material S8).

Finally, we analyzed the data from the PAAD project in the TISIDB database to reveal the expression characteristics of SUMO family members in different immune subtypes of PAAD. As shown in Figure 5I, there were significant differences in the expression levels of SUMO1/2/3 among the five immune subtypes. The expression of SUMO family members was highest in C2 subtype PAAD tissues and lowest in C3 subtype PAAD tissues.

In conclusion, the roles of SUMO family members in the immune regulation of the PAAD tumor microenvironment are complex and diverse. The high expression of SUMO1/2 may lead to the immune escape of PAAD cells, which are potential targets for the treatment of PAAD.

### Potential value of SUMO family members in clinical treatment

To explore the potential value of SUMO family members in the clinical treatment of PAAD, we first analyzed the relationships between the expression levels of SUMO family members and the expression levels of various immune checkpoint genes with cancer-promoting effects using R software (version 3.6.3) in the data from the PAAD project in the TIMER database. The results showed that there was a significant correlation of expression between SUMO family members and various immune checkpoint genes, including CD44, HHLA2, and HAVCR2 (Figure 5J) (Supplementary Material S9). These results suggest that the development of immune checkpoint inhibitors targeting these genes will be of great benefit for the treatment of PAAD. In addition, we used the GSCALite database to analyze the relationship between SUMO4 expression levels and therapeutic sensitivity to a variety of chemotherapeutics and other targeted drugs. As shown in Figure 5K, the expression level of SUMO4 was positively correlated with sensitivity to gemcitabine, paclitaxel, doxorubicin, and other drugs. In conclusion, SUMO family members can be considered promising biomarkers to predict sensitivity to PAAD treatment. Experimental studies and drug development of SUMO family members as therapeutic targets should be carried out as soon as possible, and their results are highly anticipated.

## Discussion

Members of the SUMO family play crucial roles in chromosome organization and function, genome stability, quality control of newly synthesized proteins, proteasome degradation of proteins, and DNA damage repair (Jin et al., 2017). Moreover, many cellular activities, including transcription, macromolecular assembly, protein homeostasis, transport, and signal transduction, are affected by SUMOylation (Lorente et al., 2019), such that a significant increase in the expression of SUMO family members is likely to have important impacts on cell fate. Many studies have revealed that SUMO family members have cancer-promoting effects in a variety of malignant tumors, including glioblastoma, non-small cell lung cancer, breast cancer, and liver cancer (Bawa-Khalfe and Yeh, 2010; Ke et al., 2019; Xu et al., 2020; Yang et al., 2021), but the role of SUMO family members in PAAD is still unclear, and relevant studies are scarce. In this study, the role of SUMO family members in PAAD was analyzed by bioinformatics for the first time, and the functions of SUMO family members in PAAD and the possible mechanisms involved in the occurrence and development of PAAD were comprehensively revealed from the perspectives of gene expression, gene variation, promoter methylation, immune cell infiltration, gene enrichment, PPI, and drug sensitivity.

The elevated expression level of SUMO family members is closely related to the occurrence and development of a variety of malignant tumors. Our study revealed that SUMO family members are also significantly overexpressed in PAAD. Moreover, the variations of SUMO family member gene in PAAD patients were mainly amplification and mRNA-high variants. Further investigation revealed that the high expression of SUMO family members was closely related to a more advanced clinicopathological stage and poorer prognosis in PAAD patients. These findings suggest that some potential mechanisms of action closely link the high expression of SUMO family members with the occurrence and development of PAAD.

In the investigation of related mechanisms, we first found a coexpression correlation between SUMO family members and N6-methyladenosine (M6A) methylation regulators (METTL3, METTL14, WTAP, YTHDC1, YTHDF1, YTHDF2, YTHDF3, YTHDC2, FTO, ALKBH5). In mammals, M6A is the most abundant internal mRNA modification, which is dynamic and reversible and occurs mainly in 3′ untranslated regions and near stop codons (Xu et al., 2020). Most importantly, M6A methylation regulators have been reported to be oncogenic in many studies, and their increased expression is closely related to the occurrence and development of various malignancies, including colorectal cancer, pancreatic cancer, hepatocellular carcinoma, ovarian cancer, and breast cancer (Tanabe et al., 2016; Zhang et al., 2017; Chen et al., 2019; Li J et al., 2019; Li T et al., 2019; Chang et al., 2020; Liu et al., 2020; Wang et al., 2020; Zhang et al., 2020; Cao et al., 2022). Therefore, we propose a possible oncogenic mechanism involving the high expression of SUMO family members, which increases the expression levels of M6A methylation regulators and in turn increases the oncogenic effect of M6A methylation regulators in PAAD.

Notably, we found that members of the SUMO family may be targets that drive the development and progression of PAAD after TP53 mutation. The tumor suppressor gene TP53 is located on the short arm of human chromosome 17 (Monti et al., 2020), and its expression product, the p53 protein, is the main target of tumor inactivation due to its antiproliferative effects on various physiological processes, such as the stress response and aging. However, TP53 mutation can lead to cancer in humans. TP53 mutations are most frequently missense mutations concentrated in the DNA-binding domain between codons 125 and 300 and occur in almost all types of cancer, including pancreatic cancer, ovarian cancer, esophageal cancer, colorectal cancer, head and neck cancer, laryngeal cancer and lung cancer (Olivier et al., 2010; Peltonen et al., 2010; Mogi and Kuwano, 2011; Yurgelun et al., 2015; Silwal-Pandit et al., 2017; McCubrey et al., 2022). Our study found that the expression levels of SUMO family members were significantly increased in PAAD tissues with TP53 mutations compared with PAAD with wild-type TP53, suggesting that SUMO family members may be downstream targets of mutant TP53. In addition, we found that TP53 is located in the core of the PPI network of SUMO family members, and there are strong PPIs between TP53 and several genes, including SUMO family members, which reveals the close association between TP53 and SUMO family members from another perspective. Considering that the high expression of SUMO family members in PAAD is significantly associated with the occurrence and development of PAAD and worse patient prognosis, it is reasonable to believe that the close association between TP53 mutation and the expression levels of SUMO family members may be another potential mechanism for the occurrence and development of PAAD.

To further explore the possible mechanisms of SUMO family members in PAAD carcinogenesis, we annotated the top 200 genes coexpressed with SUMO family members in the heatmap (https://www.genecards.org/). The results showed that the high expression of 35 of these genes had carcinogenic effects. These genes comprised 28 protein-coding genes, including PTMA, MRPS23, AMIGO2, and HSPA6; 5 lncRNA genes, including BICDL3P, NCRNA00152, and NEAT1; and two pseudogenes, SUMO1P3 and FTH1P3. In addition, SUMO family members generally play an activating role in the “apoptosis”, “cell cycle”, and “DNA damage response” pathways. Although apoptosis is considered to be a useful mechanism in the prevention and treatment of cancer, a study by Gabriel Achim et al. found that apoptosis can also lead to negative effects and may even promote cancer (Ichim and Tait, 2016). Moreover, cell cycle dysregulation is the basis of abnormal cell proliferation (Williams and Stoeber, 2012), and error repair after the DNA damage response is also an important cancer-promoting factor (Lord and Ashworth, 2012). More noteworthy is the activation of SUMO3/4 in the “EMT” pathway. The EMT signaling pathway is a classic cancer-promoting pathway that leads to the formation of secondary metastatic lesions by activating the mobility and invasion ability of tumor cells. It plays an oncogenic role in many tumors, including pancreatic cancer, prostate cancer, and breast cancer (Kong et al., 2021; Nowak and Bednarek, 2021; Zhao et al., 2021; Peng et al., 2022). Therefore, the coexpression characteristics of SUMO family members and various oncogenes and their roles in activating various oncogenic pathways may represent another potential mechanism for the occurrence and development of PAAD.

In addition, we analyzed the role of SUMO family members in PAAD immunity. Although immunotherapy for cancer has been shown to improve the survival rate of patients with a variety of tumors, the remission rate of PAAD patients is still very low (Ribas and Wolchok, 2018). Therefore, a comprehensive study of the interactions between tumors and immune cells will help to clarify the pathogenesis of cancer and to develop new immunotherapy strategies. We investigated the relationships between the levels of tumor-infiltrating immune cells and the expression levels of SUMO family members in PAAD. The results showed that SUMO1/2 and SUMO3 played opposite roles in PAAD immune regulation, indicating that the roles of SUMO family members in PAAD tumor immune regulation are diverse and complex. Similarly, Bolandi et al. found that B7 family members may play different roles in tumor immune regulation (Bolandi et al., 2021). This result supports our finding that different members of the SUMO family play different roles in tumor immune regulation. This phenomenon may be important for maintaining a balance between immune effectiveness and autoimmune suppression (Bolandi et al., 2021; Duan et al., 2022a; Duan et al., 2022b). More importantly, the expression level of SUMO1/2 was negatively correlated with the infiltration levels of a variety of tumor-infiltrating immune cells, including Act B, Imm B, and Tem CD8, and with most immune modulators (immune promoters, MHC molecules, chemokines, and chemokine receptors). Therefore, the high expression of SUMO1/2 may promote the immune escape of PAAD, and these factors can be used as potential targets of PAAD immunotherapy and molecular indices to predict the efficacy of immunotherapy.

Finally, we analyzed the potential value of SUMO family members in the clinical treatment of PAAD. The results showed that SUMO family members were significantly coexpressed with a variety of immune checkpoint genes, including CD44, HHLA2, and HAVCR2, that play a tumor-promoting role in malignant tumors such as PAAD, lymphoma and malignant pleural mesothelioma (https://www.genecards.org/). Therefore, research toward the development of immune checkpoint inhibitors targeting these genes is of great significance for the treatment of PAAD. In addition, we found that the expression level of SUMO4 was positively correlated with sensitivity to a variety of targeted or chemotherapeutic drugs, including gemcitabine, paclitaxel, and doxorubicin, so PAAD patients with high expression of SUMO4 may have a better response to gemcitabine and paclitaxel.

This study is the first bioinformatic analysis of SUMO family member function in PAAD, and we found that high expression of SUMO family members in PAAD is significantly correlated with specific clinicopathological features and poor prognosis in PAAD patients. Further investigation revealed that the coexpression of SUMO family members with M6A methylation regulators and a variety of oncogenes, their activating roles in a variety of oncogenic pathways, their close association with TP53 mutation status, and the negative regulatory effect of SUMO1/2 on PAAD immunity are potential mechanisms mediating the role of SUMO family members in promoting PAAD. The coexpression of SUMO family members and a variety of cancer-promoting immune checkpoint genes and the positive correlation between SUMO4 expression level and sensitivity to various targeted or chemotherapeutic drugs, including gemcitabine, paclitaxel, and doxorubicin, indicate the potential translational value of this study. However, this study also has some limitations. For example, the number of databases included in this study is somewhat low and may be inadequate. In addition, this study provides only a bioinformatic analysis of the function and mechanism of SUMO family members in the occurrence and development of PAAD. Therefore, experimental functional and mechanistic studies should be carried out as soon as possible to further confirm the cancer-promoting roles of SUMO family members in PAAD.

## Conclusion

In conclusion, bioinformatic analysis of the functions of SUMO family members in PAAD revealed that SUMO family members may promote the occurrence and development of PAAD and can be used as new biomarkers and therapeutic targets for PAAD patients.

## Data Availability

The datasets presented in this study can be found in online repositories. The names of the repository/repositories and accession number(s) can be found in the article/Supplementary Material.

## Supplementary material

Supplementary Figure S1 Expression characteristics of SUMO3/4 at the single-cell level in PAAD. **(A)** The cell group; **(B)** The single-cell level of SUMO3; **(C)** The single-cell level of SUMO4.

Supplementary Figure S2 Top 50 genes co-expressed with SUMO3/4. **(A)** Top 50 genes co-expressed with SUMO3; **(B)** Top 50 genes co-expressed with SUMO4.

Supplementary Figure S3 The GO enrichment of the CC terms and MF terms of the SUMO family and its 400 co-expressed genes. **(A)** CC terms; **(B)** MF terms.

Supplementary Figure S4 Relationship between SUMO1/2/3 expression levels and infiltration levels of multiple tumor infiltrating immune cells. **(A)** SUMO1; **(B)** SUMO2; **(C)** SUMO3.

## Abbreviations

PAAD, pancreatic adenocarcinoma; SUMO, small ubiquitin-like modifier; M6A, N6-methyladenosine; PPI, protein–protein interaction; TCGA, The Cancer Genome Atlas; GTEx, Genotype-Tissue Expression; STAD, stomach adenocarcinoma; BRCA, breast invasive carcinoma; KICH, kidney chromophobe; LAML, acute myeloid leukemia; COAD, colon adenocarcinoma; LIHC, liver hepatocellular carcinoma; OV, ovarian serous cystadenocarcinoma; OS, overall survival; RFS, recurrence-free survival; GO, Gene Ontology; KEGG, Kyoto Encyclopedia of Genes and Genomes; BP, biological process; CC, cellular components; MF, molecular function; EMT, epithelial–mesenchymal transition.
