# Supplementary figures and images for: Prognostic value, immune signature and molecular mechanisms of the SUMO family in pancreatic adenocarcinoma
Source: Front Mol Biosci. 2022 Dec 15;9:1096679. doi: 10.3389/fmolb.2022.1096679 (PMC9798011; doi:10.3389/fmolb.2022.1096679)

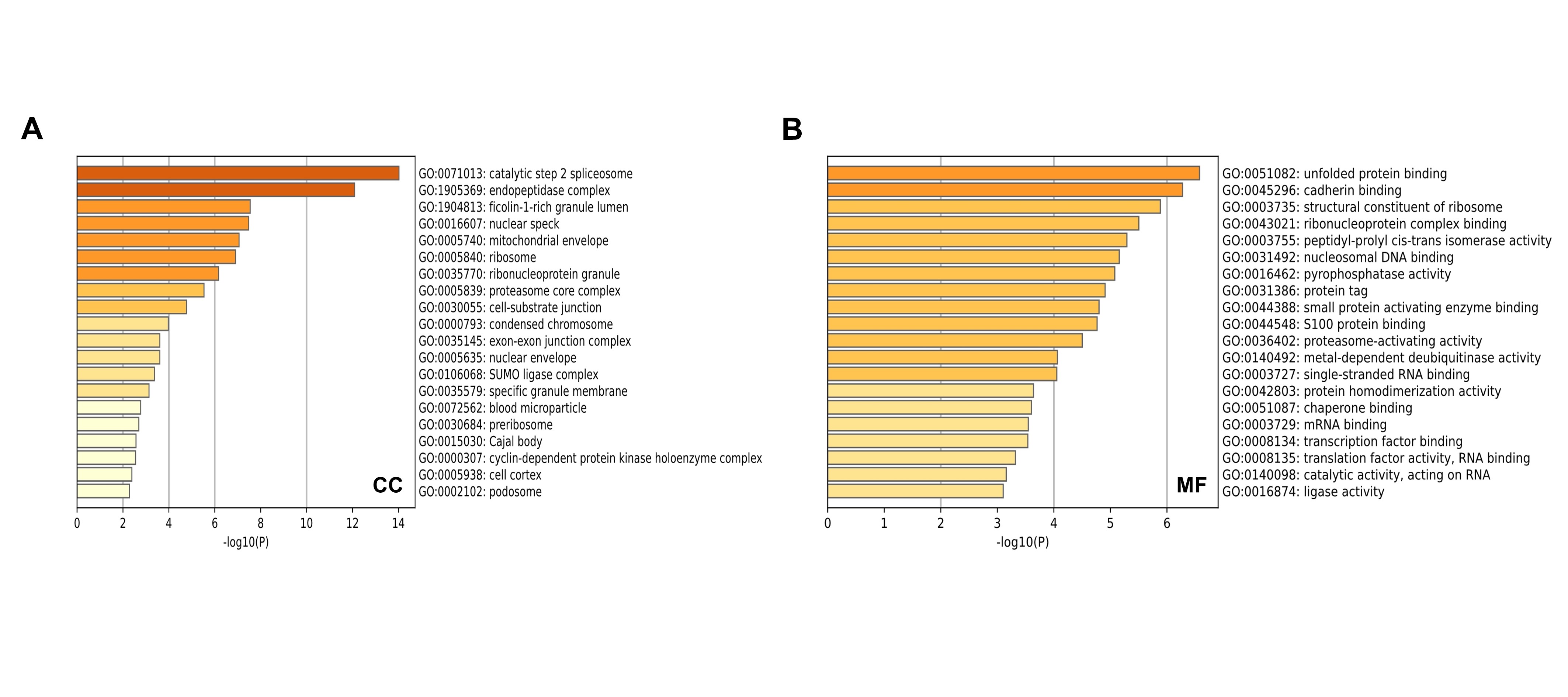

Supplement: Supplementary file 2 [file Image3.JPEG]

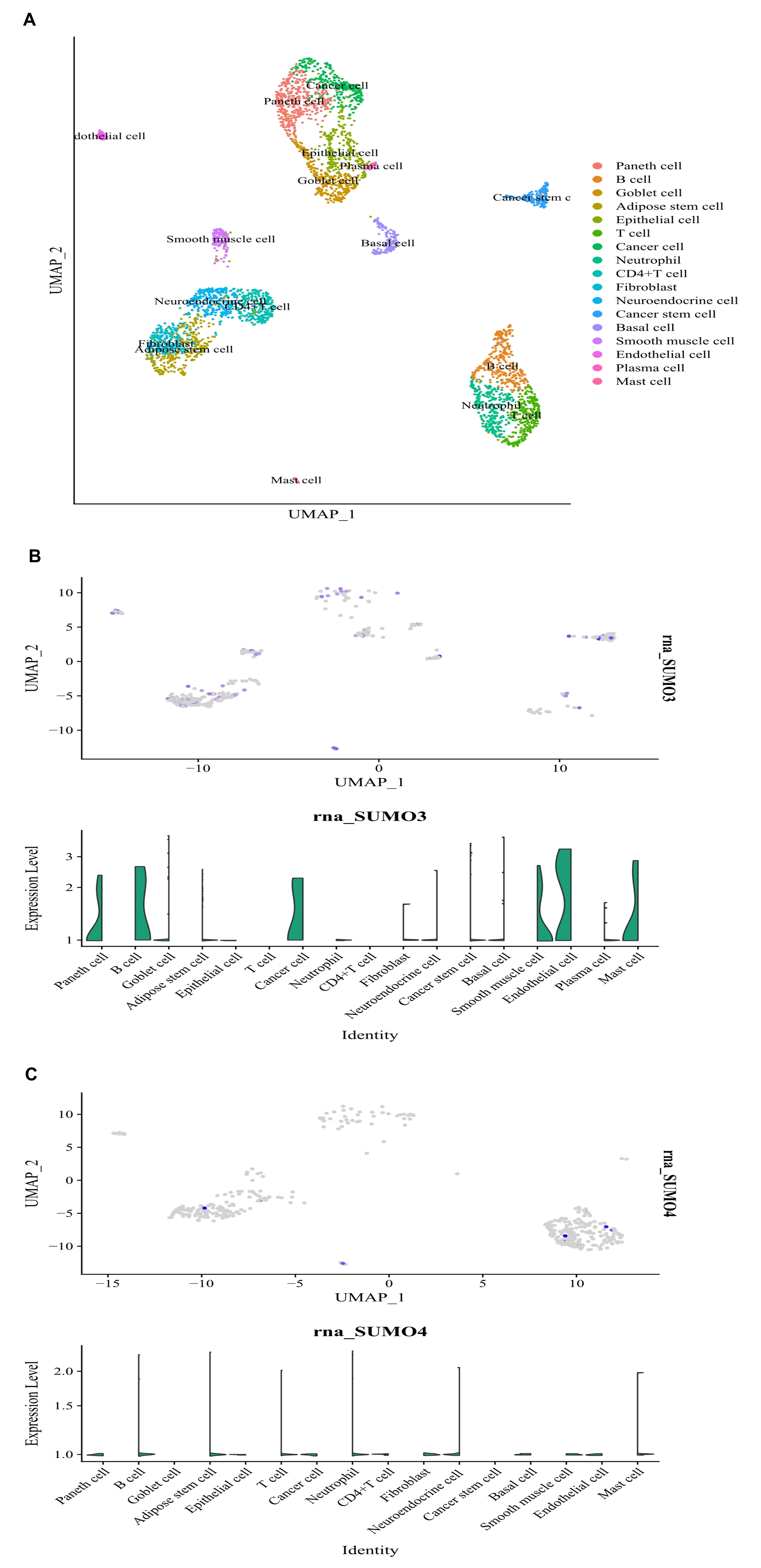

Supplement: Supplementary file 6 [file Image1.JPEG]

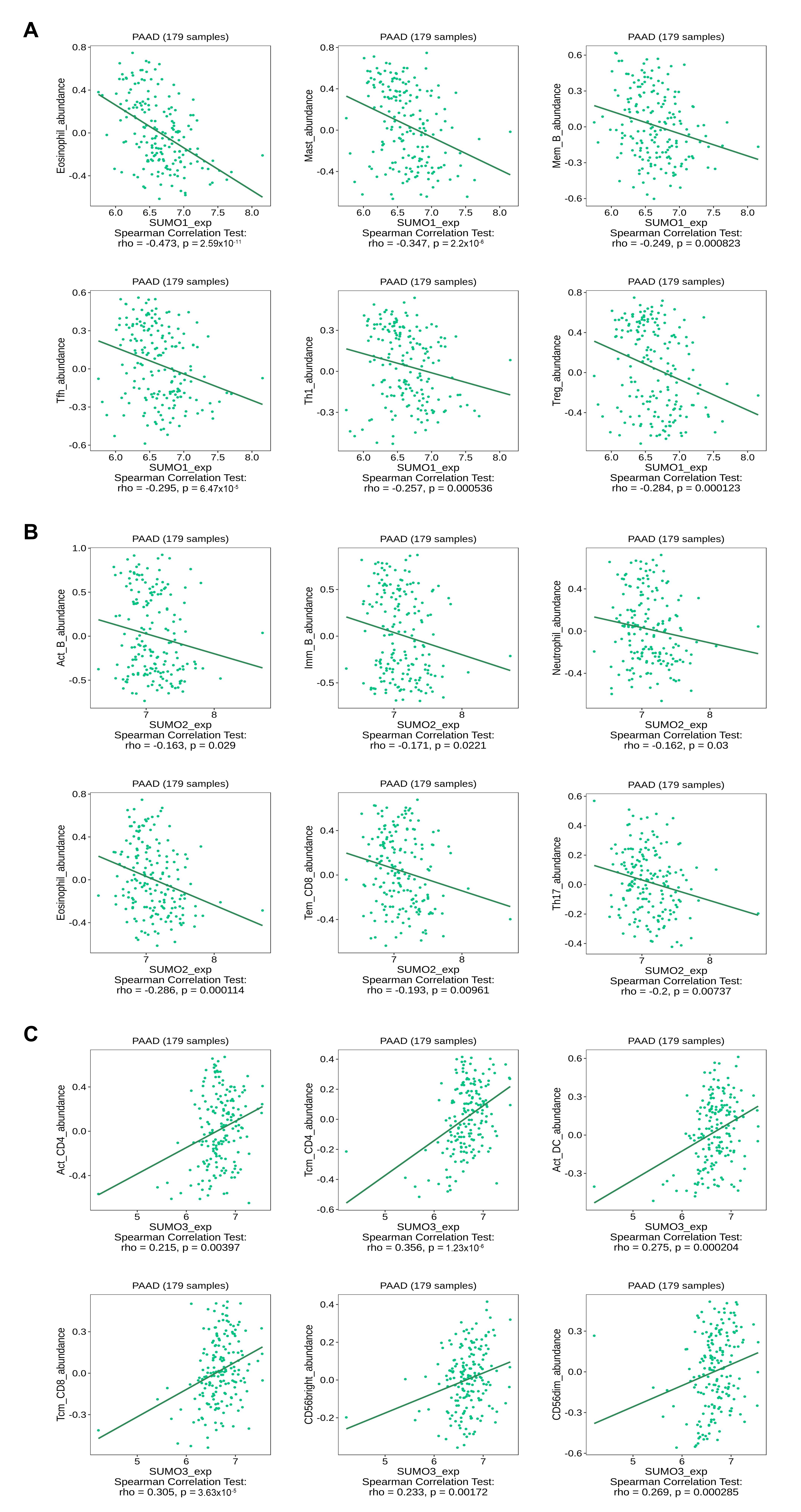

Supplement: Supplementary file 7 [file Image4.JPEG]

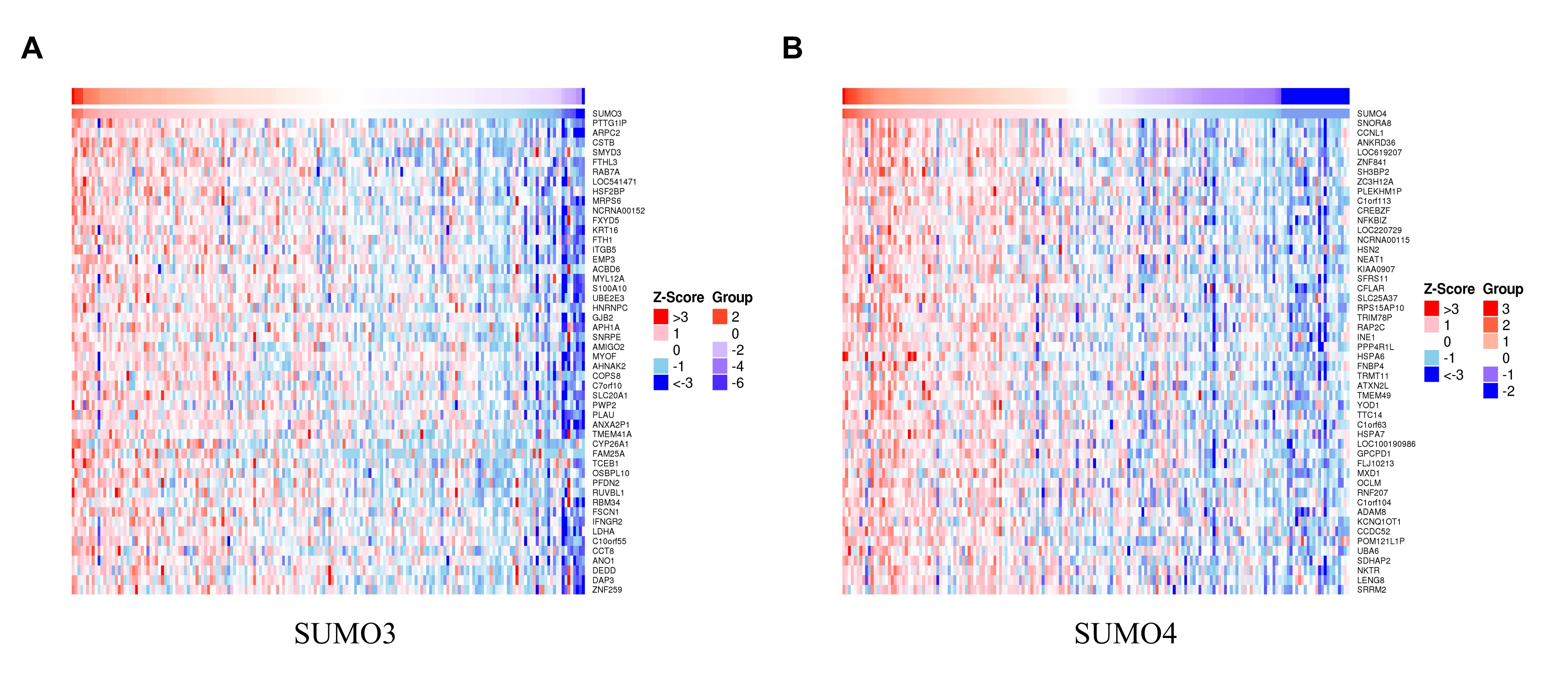

Supplement: Supplementary file 8 [file Image2.JPEG]
