# Supplementary material for: Prognostic value, immune signature and molecular mechanisms of the SUMO family in pancreatic adenocarcinoma
Source: Front Mol Biosci. 2022 Dec 15;9:1096679. doi: 10.3389/fmolb.2022.1096679 (PMC9798011; doi:10.3389/fmolb.2022.1096679)
Supplement: Supplementary file 4 [file Table1.DOCX]

| Characteristic  Relationship between the SUMO family expression and clinicopathological characteristics of PAAD patients. | Low expression of SUMO1 | High expression of SUMO1 | p |
| --- | --- | --- | --- |
| n | 89 | 89 |  |
| T stage, n (%) |  |  | 0.927 |
| T1 | 3 (1.7%) | 4 (2.3%) |  |
| T2 | 13 (7.4%) | 11 (6.2%) |  |
| T3 | 71 (40.3%) | 71 (40.3%) |  |
| T4 | 1 (0.6%) | 2 (1.1%) |  |
| N stage, n (%) |  |  | 1.000 |
| N0 | 25 (14.5%) | 25 (14.5%) |  |
| N1 | 61 (35.3%) | 62 (35.8%) |  |
| M stage, n (%) |  |  | 0.363 |
| M0 | 39 (46.4%) | 40 (47.6%) |  |
| M1 | 1 (1.2%) | 4 (4.8%) |  |
| Pathologic stage, n (%) |  |  | 0.526 |
| Stage I | 10 (5.7%) | 11 (6.3%) |  |
| Stage II | 76 (43.4%) | 70 (40%) |  |
| Stage III | 1 (0.6%) | 2 (1.1%) |  |
| Stage IV | 1 (0.6%) | 4 (2.3%) |  |
| Radiation therapy, n (%) |  |  | 0.578 |
| No | 58 (35.6%) | 60 (36.8%) |  |
| Yes | 25 (15.3%) | 20 (12.3%) |  |
| Primary therapy outcome, n (%) |  |  | 0.222 |
| PD | 19 (13.7%) | 30 (21.6%) |  |
| SD | 4 (2.9%) | 5 (3.6%) |  |
| PR | 7 (5%) | 3 (2.2%) |  |
| CR | 38 (27.3%) | 33 (23.7%) |  |
| Gender, n (%) |  |  | 0.880 |
| Female | 39 (21.9%) | 41 (23%) |  |
| Male | 50 (28.1%) | 48 (27%) |  |
| Race, n (%) |  |  | 0.740 |
| Asian | 5 (2.9%) | 6 (3.4%) |  |
| Black or African American | 4 (2.3%) | 2 (1.1%) |  |
| White | 77 (44.3%) | 80 (46%) |  |
| Age, n (%) |  |  | 0.548 |
| <=65 | 44 (24.7%) | 49 (27.5%) |  |
| >65 | 45 (25.3%) | 40 (22.5%) |  |
| Residual tumor, n (%) |  |  | 0.592 |
| R0 | 56 (34.1%) | 51 (31.1%) |  |
| R1 | 23 (14%) | 29 (17.7%) |  |
| R2 | 2 (1.2%) | 3 (1.8%) |  |
| Histologic grade, n (%) |  |  | 0.062 |
| G1 | 21 (11.9%) | 10 (5.7%) |  |
| G2 | 43 (24.4%) | 52 (29.5%) |  |
| G3 | 22 (12.5%) | 26 (14.8%) |  |
| G4 | 2 (1.1%) | 0 (0%) |  |
| Anatomic neoplasm subdivision, n (%) |  |  | 0.857 |
| Head of Pancreas | 70 (39.3%) | 68 (38.2%) |  |
| Other | 19 (10.7%) | 21 (11.8%) |  |
| Smoker, n (%) |  |  | 0.974 |
| No | 34 (23.6%) | 31 (21.5%) |  |
| Yes | 40 (27.8%) | 39 (27.1%) |  |
| Alcohol history, n (%) |  |  | 1.000 |
| No | 33 (19.9%) | 32 (19.3%) |  |
| Yes | 52 (31.3%) | 49 (29.5%) |  |
| History of diabetes, n (%) |  |  | 0.916 |
| No | 57 (39%) | 51 (34.9%) |  |
| Yes | 19 (13%) | 19 (13%) |  |
| History of chronic pancreatitis, n (%) |  |  | 0.194 |
| No | 69 (48.9%) | 59 (41.8%) |  |
| Yes | 4 (2.8%) | 9 (6.4%) |  |
| Family history of cancer, n (%) |  |  | 0.205 |
| No | 21 (19.1%) | 26 (23.6%) |  |
| Yes | 37 (33.6%) | 26 (23.6%) |  |
| Age, mean ± SD | 65.03 ± 10.54 | 64.46 ± 11.11 | 0.724 |

| Characteristic | Low expression of SUMO2 | High expression of SUMO2 | p |
| --- | --- | --- | --- |
| n | 89 | 89 |  |
| T stage, n (%) |  |  | 0.651 |
| T1 | 5 (2.8%) | 2 (1.1%) |  |
| T2 | 13 (7.4%) | 11 (6.2%) |  |
| T3 | 70 (39.8%) | 72 (40.9%) |  |
| T4 | 1 (0.6%) | 2 (1.1%) |  |
| N stage, n (%) |  |  | 0.905 |
| N0 | 26 (15%) | 24 (13.9%) |  |
| N1 | 61 (35.3%) | 62 (35.8%) |  |
| M stage, n (%) |  |  | 0.064 |
| M0 | 37 (44%) | 42 (50%) |  |
| M1 | 0 (0%) | 5 (6%) |  |
| Pathologic stage, n (%) |  |  | 0.067 |
| Stage I | 13 (7.4%) | 8 (4.6%) |  |
| Stage II | 75 (42.9%) | 71 (40.6%) |  |
| Stage III | 1 (0.6%) | 2 (1.1%) |  |
| Stage IV | 0 (0%) | 5 (2.9%) |  |
| Radiation therapy, n (%) |  |  | 0.040 |
| No | 53 (32.5%) | 65 (39.9%) |  |
| Yes | 29 (17.8%) | 16 (9.8%) |  |
| Primary therapy outcome, n (%) |  |  | 0.584 |
| PD | 20 (14.4%) | 29 (20.9%) |  |
| SD | 5 (3.6%) | 4 (2.9%) |  |
| PR | 6 (4.3%) | 4 (2.9%) |  |
| CR | 36 (25.9%) | 35 (25.2%) |  |
| Gender, n (%) |  |  | 0.651 |
| Female | 42 (23.6%) | 38 (21.3%) |  |
| Male | 47 (26.4%) | 51 (28.7%) |  |
| Race, n (%) |  |  | 0.329 |
| Asian | 5 (2.9%) | 6 (3.4%) |  |
| Black or African American | 5 (2.9%) | 1 (0.6%) |  |
| White | 78 (44.8%) | 79 (45.4%) |  |
| Age, n (%) |  |  | 0.368 |
| <=65 | 43 (24.2%) | 50 (28.1%) |  |
| >65 | 46 (25.8%) | 39 (21.9%) |  |
| Residual tumor, n (%) |  |  | 0.833 |
| R0 | 52 (31.7%) | 55 (33.5%) |  |
| R1 | 27 (16.5%) | 25 (15.2%) |  |
| R2 | 2 (1.2%) | 3 (1.8%) |  |
| Histologic grade, n (%) |  |  | 0.465 |
| G1 | 19 (10.8%) | 12 (6.8%) |  |
| G2 | 46 (26.1%) | 49 (27.8%) |  |
| G3 | 21 (11.9%) | 27 (15.3%) |  |
| G4 | 1 (0.6%) | 1 (0.6%) |  |
| Smoker, n (%) |  |  | 1.000 |
| No | 32 (22.2%) | 33 (22.9%) |  |
| Yes | 40 (27.8%) | 39 (27.1%) |  |
| Anatomic neoplasm subdivision, n (%) |  |  | 0.369 |
| Head of Pancreas | 72 (40.4%) | 66 (37.1%) |  |
| Other | 17 (9.6%) | 23 (12.9%) |  |
| Alcohol history, n (%) |  |  | 1.000 |
| No | 34 (20.5%) | 31 (18.7%) |  |
| Yes | 52 (31.3%) | 49 (29.5%) |  |
| History of diabetes, n (%) |  |  | 0.640 |
| No | 53 (36.3%) | 55 (37.7%) |  |
| Yes | 21 (14.4%) | 17 (11.6%) |  |
| History of chronic pancreatitis, n (%) |  |  | 1.000 |
| No | 64 (45.4%) | 64 (45.4%) |  |
| Yes | 6 (4.3%) | 7 (5%) |  |
| Family history of cancer, n (%) |  |  | 0.247 |
| No | 20 (18.2%) | 27 (24.5%) |  |
| Yes | 35 (31.8%) | 28 (25.5%) |  |
| Age, mean ± SD | 66.34 ± 10.64 | 63.16 ± 10.78 | 0.049 |

| Characteristic | Low expression of SUMO3 | High expression of SUMO3 | p |
| --- | --- | --- | --- |
| n | 89 | 89 |  |
| T stage, n (%) |  |  | 0.290 |
| T1 | 4 (2.3%) | 3 (1.7%) |  |
| T2 | 16 (9.1%) | 8 (4.5%) |  |
| T3 | 66 (37.5%) | 76 (43.2%) |  |
| T4 | 1 (0.6%) | 2 (1.1%) |  |
| N stage, n (%) |  |  | 0.754 |
| N0 | 26 (15%) | 24 (13.9%) |  |
| N1 | 59 (34.1%) | 64 (37%) |  |
| M stage, n (%) |  |  | 0.068 |
| M0 | 36 (42.9%) | 43 (51.2%) |  |
| M1 | 0 (0%) | 5 (6%) |  |
| Pathologic stage, n (%) |  |  | 0.036 |
| Stage I | 14 (8%) | 7 (4%) |  |
| Stage II | 72 (41.1%) | 74 (42.3%) |  |
| Stage III | 1 (0.6%) | 2 (1.1%) |  |
| Stage IV | 0 (0%) | 5 (2.9%) |  |
| Radiation therapy, n (%) |  |  | 0.837 |
| No | 59 (36.2%) | 59 (36.2%) |  |
| Yes | 24 (14.7%) | 21 (12.9%) |  |
| Primary therapy outcome, n (%) |  |  | 0.380 |
| PD | 23 (16.5%) | 26 (18.7%) |  |
| SD | 7 (5%) | 2 (1.4%) |  |
| PR | 6 (4.3%) | 4 (2.9%) |  |
| CR | 36 (25.9%) | 35 (25.2%) |  |
| Gender, n (%) |  |  | 0.880 |
| Female | 39 (21.9%) | 41 (23%) |  |
| Male | 50 (28.1%) | 48 (27%) |  |
| Age, n (%) |  |  | 1.000 |
| <=65 | 47 (26.4%) | 46 (25.8%) |  |
| >65 | 42 (23.6%) | 43 (24.2%) |  |
| Race, n (%) |  |  | 0.244 |
| Asian | 8 (4.6%) | 3 (1.7%) |  |
| Black or African American | 2 (1.1%) | 4 (2.3%) |  |
| White | 77 (44.3%) | 80 (46%) |  |
| Residual tumor, n (%) |  |  | 0.916 |
| R0 | 53 (32.3%) | 54 (32.9%) |  |
| R1 | 27 (16.5%) | 25 (15.2%) |  |
| R2 | 3 (1.8%) | 2 (1.2%) |  |
| Histologic grade, n (%) |  |  | < 0.001 |
| G1 | 24 (13.6%) | 7 (4%) |  |
| G2 | 43 (24.4%) | 52 (29.5%) |  |
| G3 | 18 (10.2%) | 30 (17%) |  |
| G4 | 2 (1.1%) | 0 (0%) |  |
| Anatomic neoplasm subdivision, n (%) |  |  | 0.369 |
| Head of Pancreas | 72 (40.4%) | 66 (37.1%) |  |
| Other | 17 (9.6%) | 23 (12.9%) |  |
| Smoker, n (%) |  |  | 0.001 |
| No | 44 (30.6%) | 21 (14.6%) |  |
| Yes | 31 (21.5%) | 48 (33.3%) |  |
| Alcohol history, n (%) |  |  | 0.561 |
| No | 36 (21.7%) | 29 (17.5%) |  |
| Yes | 50 (30.1%) | 51 (30.7%) |  |
| History of diabetes, n (%) |  |  | 0.582 |
| No | 55 (37.7%) | 53 (36.3%) |  |
| Yes | 22 (15.1%) | 16 (11%) |  |
| History of chronic pancreatitis, n (%) |  |  | 1.000 |
| No | 68 (48.2%) | 60 (42.6%) |  |
| Yes | 7 (5%) | 6 (4.3%) |  |
| Family history of cancer, n (%) |  |  | 0.378 |
| No | 22 (20%) | 25 (22.7%) |  |
| Yes | 36 (32.7%) | 27 (24.5%) |  |
| Age, mean ± SD | 65.22 ± 10.58 | 64.27 ± 11.05 | 0.557 |

| Characteristic | Low expression of SUMO4 | High expression of SUMO4 | p |
| --- | --- | --- | --- |
| n | 89 | 89 |  |
| T stage, n (%) |  |  | 0.201 |
| T1 | 6 (3.4%) | 1 (0.6%) |  |
| T2 | 13 (7.4%) | 11 (6.2%) |  |
| T3 | 67 (38.1%) | 75 (42.6%) |  |
| T4 | 1 (0.6%) | 2 (1.1%) |  |
| N stage, n (%) |  |  | 0.375 |
| N0 | 28 (16.2%) | 22 (12.7%) |  |
| N1 | 58 (33.5%) | 65 (37.6%) |  |
| M stage, n (%) |  |  | 1.000 |
| M0 | 37 (44%) | 42 (50%) |  |
| M1 | 2 (2.4%) | 3 (3.6%) |  |
| Pathologic stage, n (%) |  |  | 0.163 |
| Stage I | 15 (8.6%) | 6 (3.4%) |  |
| Stage II | 69 (39.4%) | 77 (44%) |  |
| Stage III | 1 (0.6%) | 2 (1.1%) |  |
| Stage IV | 2 (1.1%) | 3 (1.7%) |  |
| Radiation therapy, n (%) |  |  | 0.690 |
| No | 61 (37.4%) | 57 (35%) |  |
| Yes | 21 (12.9%) | 24 (14.7%) |  |
| Primary therapy outcome, n (%) |  |  | 0.847 |
| PD | 23 (16.5%) | 26 (18.7%) |  |
| SD | 5 (3.6%) | 4 (2.9%) |  |
| PR | 4 (2.9%) | 6 (4.3%) |  |
| CR | 37 (26.6%) | 34 (24.5%) |  |
| Gender, n (%) |  |  | 0.451 |
| Female | 43 (24.2%) | 37 (20.8%) |  |
| Male | 46 (25.8%) | 52 (29.2%) |  |
| Race, n (%) |  |  | 0.852 |
| Asian | 5 (2.9%) | 6 (3.4%) |  |
| Black or African American | 2 (1.1%) | 4 (2.3%) |  |
| White | 79 (45.4%) | 78 (44.8%) |  |
| Age, n (%) |  |  | 1.000 |
| <=65 | 47 (26.4%) | 46 (25.8%) |  |
| >65 | 42 (23.6%) | 43 (24.2%) |  |
| Residual tumor, n (%) |  |  | 0.172 |
| R0 | 59 (36%) | 48 (29.3%) |  |
| R1 | 21 (12.8%) | 31 (18.9%) |  |
| R2 | 2 (1.2%) | 3 (1.8%) |  |
| Histologic grade, n (%) |  |  | 0.212 |
| G1 | 20 (11.4%) | 11 (6.2%) |  |
| G2 | 47 (26.7%) | 48 (27.3%) |  |
| G3 | 20 (11.4%) | 28 (15.9%) |  |
| G4 | 1 (0.6%) | 1 (0.6%) |  |
| Anatomic neoplasm subdivision, n (%) |  |  | 0.857 |
| Head of Pancreas | 70 (39.3%) | 68 (38.2%) |  |
| Other | 19 (10.7%) | 21 (11.8%) |  |
| Smoker, n (%) |  |  | 0.144 |
| No | 39 (27.1%) | 26 (18.1%) |  |
| Yes | 33 (22.9%) | 46 (31.9%) |  |
| Alcohol history, n (%) |  |  | 0.571 |
| No | 34 (20.5%) | 31 (18.7%) |  |
| Yes | 47 (28.3%) | 54 (32.5%) |  |
| History of diabetes, n (%) |  |  | 0.516 |
| No | 54 (37%) | 54 (37%) |  |
| Yes | 16 (11%) | 22 (15.1%) |  |
| History of chronic pancreatitis, n (%) |  |  | 0.542 |
| No | 66 (46.8%) | 62 (44%) |  |
| Yes | 5 (3.5%) | 8 (5.7%) |  |
| Family history of cancer, n (%) |  |  | 0.544 |
| No | 26 (23.6%) | 21 (19.1%) |  |
| Yes | 30 (27.3%) | 33 (30%) |  |
| Age, mean ± SD | 64.85 ± 10.7 | 64.64 ± 10.95 | 0.896 |
