# Supplementary material for: Prognostic value, immune signature and molecular mechanisms of the SUMO family in pancreatic adenocarcinoma
Source: Front Mol Biosci. 2022 Dec 15;9:1096679. doi: 10.3389/fmolb.2022.1096679 (PMC9798011; doi:10.3389/fmolb.2022.1096679)
Supplement: Supplementary file 10 [file Table5.DOCX]

Enrichment analysis of the SUMO family and its 400 co-expressed genes.

ExpandCategory TermID Description LogP

GO Biological Processes GO:0022613 ribonucleoprotein complex biogenesis -13.73931

GO Biological Processes GO:0044265 cellular macromolecule catabolic process -9.54833

GO Biological Processes GO:0042254 ribosome biogenesis -7.66418

GO Biological Processes GO:0006412 translation -7.59220

GO Biological Processes GO:0006457 protein folding -6.42314

GO Biological Processes GO:0016925 protein sumoylation -5.8447

GO Biological Processes GO:0006913 nucleocytoplasmic transport -5.81914

GO Biological Processes GO:0044403 biological process involved in symbiotic interaction -5.51914

GO Biological Processes GO:0030162 regulation of proteolysis -5.44125

GO Biological Processes GO:0006458 'de novo' protein folding -5.0926

GO Biological Processes GO:0043484 regulation of RNA splicing -4.98711

GO Biological Processes GO:0006402 mRNA catabolic process -4.7729

GO Biological Processes GO:0009141 nucleoside triphosphate metabolic process -4.44211

GO Biological Processes GO:0033365 protein localization to organelle -4.38622

GO Biological Processes GO:0010755 regulation of plasminogen activation -4.2964

GO Biological Processes GO:0051099 positive regulation of binding -4.29510

GO Biological Processes GO:0046654 tetrahydrofolate biosynthetic process -4.0633

GO Biological Processes GO:0140694 non-membrane-bounded organelle assembly -3.9841

GO Biological Processes GO:0070527 platelet aggregation -3.8355

GO Biological Processes GO:1903320 regulation of protein modification by small protein conjugation or removal -3.66511

KEGG Pathway hsa03050 Proteasome -12.79812

KEGG Pathway hsa03040 Spliceosome -12.77818

KEGG Pathway hsa03013 Nucleocytoplasmic transport -7.19411

KEGG Pathway hsa05418 Fluid shear stress and atherosclerosis -6.07711

KEGG Pathway hsa05132 Salmonella infection -4.35712

KEGG Pathway hsa04120 Ubiquitin mediated proteolysis -3.5378

KEGG Pathway hsa03010 Ribosome -3.2278

KEGG Pathway hsa00670 One carbon pool by folate -2.7993

KEGG Pathway hsa03060 Protein export -2.6193

KEGG Pathway hsa04217 Necroptosis -2.5447

KEGG Pathway hsa05134 Legionellosis -2.3414

KEGG Pathway hsa03008 Ribosome biogenesis in eukaryotes -2.0245

GO Cellular Components GO:0071013 catalytic step 2 spliceosome -14.027

GO Cellular Components GO:1905369 endopeptidase complex -12.093

GO Cellular Components GO:1904813 ficolin-1-rich granule lumen -7.543

GO Cellular Components GO:0016607 nuclear speck -7.478

GO Cellular Components GO:0005740 mitochondrial envelope -7.059

GO Cellular Components GO:0005840 ribosome -6.904

GO Cellular Components GO:0035770 ribonucleoprotein granule -6.163

GO Cellular Components GO:0005839 proteasome core complex -5.53

GO Cellular Components GO:0030055 cell-substrate junction -4.767

GO Cellular Components GO:0000793 condensed chromosome -3.975

GO Cellular Components GO:0035145 exon-exon junction complex -3.60

GO Cellular Components GO:0005635 nuclear envelope -3.604

GO Cellular Components GO:0106068 SUMO ligase complex -3.373

GO Cellular Components GO:0035579 specific granule membran e-3.13

GO Cellular Components GO:0072562 blood microparticle -2.770

GO Cellular Components GO:0030684 preribosome -2.689

GO Cellular Components GO:0015030 Cajal body -2.568

GO Cellular Components GO:0000307 cyclin-dependent protein kinase holoenzyme complex -2.548

GO Cellular Components GO:0005938 cell cortex -2.3861

GO Cellular Components GO:0002102 podosome -2.28

GO Molecular Functions GO:0051082 unfolded protein binding -6.5761

GO Molecular Functions GO:0045296 cadherin binding -6.2751

GO Molecular Functions GO:0003735 structural constituent of ribosome -5.8831

GO Molecular Functions GO:0043021 ribonucleoprotein complex binding -5.5021

GO Molecular Functions GO:0003755 peptidyl-prolyl cis-trans isomerase activity -5.291

GO Molecular Functions GO:0031492 nucleosomal DNA binding -5.157

GO Molecular Functions GO:0016462 pyrophosphatase activity -5.0772

GO Molecular Functions GO:0031386 protein tag -4.907

GO Molecular Functions GO:0044388 small protein activating enzyme binding -4.799

GO Molecular Functions GO:0044548 S100 protein binding -4.765

GO Molecular Functions GO:0036402 proteasome-activating activity -4.502

GO Molecular Functions GO:0140492 metal-dependent deubiquitinase activity -4.063

GO Molecular Functions GO:0003727 single-stranded RNA binding -4.052

GO Molecular Functions GO:0042803 protein homodimerization activity -3.6392

GO Molecular Functions GO:0051087 chaperone binding -3.603

GO Molecular Functions GO:0003729 mRNA binding -3.5481

GO Molecular Functions GO:0008134 transcription factor binding -3.5391

GO Molecular Functions GO:000813 5translation factor activity, RNA binding -3.320

GO Molecular Functions GO:0140098 catalytic activity, acting on RNA -3.1591

GO Molecular Functions GO:0016874 ligase activity -3.10
